# Magnetic Water

**Published:** 1872-10

**Authors:** 


### MAGNETIC WATER.

Many people have great faith in the efficacy of mineral waters in the cure of disease. Any person who is fortunate enough to find a spring whose water is sufficiently nasty to the taste, to give it an appearance of being *medicinal*, is certain to reap a rich harvest from the ailing, provided he will erect a suitable building for the accommodation of all who may apply for an opportunity to slack their thirst at the healing fount. As mineral springs are not sufficiently abundant, enterprising gentlemen, desirous of supplying the demand for such resorts, and at the same time "turn an honest penny," are just now industriously engaged in unearthing "magnetic springs," and are meeting with their full share of public patronage. What surprises us is, that scientific men should lend their aid to such fraudulent practices. A friend has handed us a bottle of magnetic spring water, from a somewhat celebrated spring in a western State, upon the label of which we see an analysis of the water, and a voluntary statement of the Professor of Chemistry, to the effect that he is unable to account for the current of electricity or magnetism which the water contains. Any school boy in this part of the country, is familiar with the fact, that when a bar of iron, or an iron tube, is found imbedded vertically in the ground, that by rubbing a blade of your pocket knife or other steel instrument upon it, it at once becomes magnetized, so that a needle can be raised by it. The tubular, or driven well is very common in this city, and we perhaps are imparting no secret when we assure the possessors of all such wells, that they have "magnetic springs" equal to the best of them, as is shown in the fact that small steel articles can be magnetized by rubbing them upon the iron tube where it emerges from the ground. Try it and see, but do not claim that the tube is rendered magnetic by reason of the magnetism in the water being imparted to it, for such is not the fact—the magnetism comes from the earth, and is present in all places.

